# The Microstructural Evolution of Cu-Sn-P Alloy during Hot Deformation Process

**DOI:** 10.3390/ma15134501

**Published:** 2022-06-26

**Authors:** Junsheng Zhao, Limin Zhang, Fengming Du, Xia Yuan, Pengfei Wang

**Affiliations:** 1 School of Mechanical Engineering, North University of China, Taiyuan 030051, China; yuaxia@nuc.edu.cn; 2 North Engine Research Institute, Tianjin 300400, China; tju.zlmwan@aliyun.com; 3 International Shipping Research Institute, Gongqing Institute of Science and Technology, Jiujiang 332020, China; dfm121212@163.com

**Keywords:** Cu-Sn-P alloy, hot deformation, grain orientation, texture, dislocation

## Abstract

The microstructure evolution of Cu-Sn-P alloy subjected to hot deformation was researched through electron backscatter diffraction (EBSD) and transmission electron microscope (TEM) in the present study. The results indicated that after hot deformation, grains perpendicular to the force direction were elongated, and mostly became deformed grains, and then exhibited an obvious hardening effect. The Cu-Sn-P alloy could be strain hardened during hot deformation, but, with recrystallization, a softening effect occurred. Changes in dislocation density, textures, and grain sizes play different roles in flow stress behaviors of Cu-Sn-P alloy, and the dislocation density has a more evident effect at low temperature. However, with increase in temperature, recrystallization softening gradually dominates. Low-angle grain boundaries (LABs) account for the majority of hot deformed microstructures of Cu-Sn-P alloy. High dislocation densities in these zones make it easy to initiate the dislocation slipping systems. Deformation is realized through dislocation slipping and the slipping of edge dislocation pairs. The dislocation pile-up zones have high distortion energies, and, thus, elements of diffusion and recrystallization nucleation can occur easily. At different temperatures, the maximum polar density of textures gradually increases, and there are preferred orientations of grains. At 500 °C, stacking faults accumulate and promote the growth of twins. The twin growth direction is mainly determined by the migration of high-angle grain boundaries (HABs) and the clustering of high-stress zones.

## 1. Introduction

The Cu-Sn-P alloy is one of the earliest non-ferrous alloys used by humans [1,2,3]. To obtain good comprehensive performance to meet applications, P, Bi, Sb, and other elements are added into the Cu-Sn-P alloy, resulting in the formation of Cu-Sn-P alloy that possesses good mechanical properties [4]. Hot deformation is a common method for fabricating the Cu-Sn-P alloy [5,6,7]. Generally, in hot deformation, dynamic recovery occurs first, which causes the annihilation of dislocations and, thus, leads to the formation of sub-grains. Dynamic recrystallization (DRX) softening is triggered when dislocation density accumulates to a certain level [8]. Compared with the common Cu-Sn-P alloy, more alloying elements added to form the Cu-Sn-P alloy, not only make it stronger and harder, but also make it easier for it to crack during the deformation process. A. Gholinia investigated the hot deformed microstructures of annealed and cold-rolled tin bronze through 3D electron backscatter diffraction analysis [9]. It is found that recrystallization in Cu-Sn-P alloy usually starts at the point where the crystal lattices twist. New grains form in the zone where twins appear, and DRX nucleation is initiated. Grains grow along the previous grain boundary, forming different series of recrystallized groups. Jun Hui et al. showed that in the hot deformed microstructure of a Cu alloy, the combined effect of high temperature and plastic deformation caused newly produced low-angle grain boundaries (LABs) to gradually merge and migrate into high-angle grain boundaries (HABs), resulting in an obvious increase in amounts of HABs and recrystallized grains [4]. In terms of the deformation mechanism, Darling K.A. et al. discovered a representative mechanism, characterized by “double-peak microstructures of grain size” that could regulate the strength and plasticity [10]. They concluded that control of grain sizes can improve the capacity to store dislocations. Similar efforts by Sang-Heon Kim et al. shed light on a new deformation mechanism in which B_2_ particles, that are immune to the cutting effect of slipping dislocations, were introduced to promote more effective slipping of dislocations during deformation [11]. T.H. Fang et al. indicated that nano twins, introduced by precipitation, can make the alloy more processable [12].

In understanding the hot deformation mechanism of the Cu-Sn-P alloy, the grain size, dislocation evolution, and the coordination of twins need to be considered. Therefore, this study analyzed the deformation characteristics and microstructures of the Cu-Sn-P alloy under hot deformation. An isothermal compression experiment of a cylindrical specimen at a constant strain rate was conducted to investigate the DRX and microstructural evolution of the Cu-Sn-P alloy, which revealed its deformation mechanism and microstructural transformation pattern at different temperatures.

## 2. Experimental Procedures

**Figure 1:**
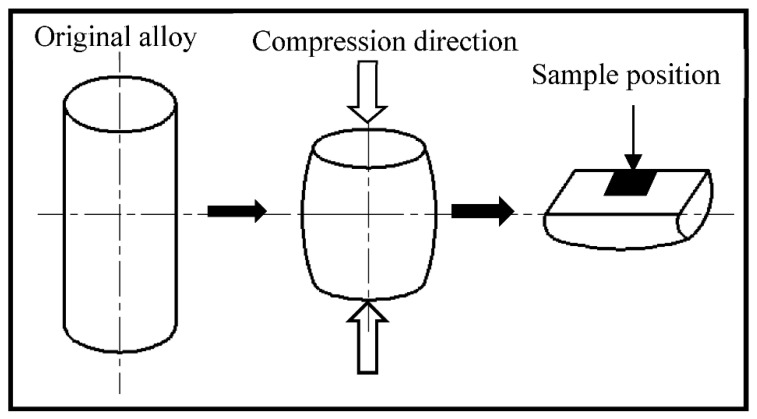
The schematic of thermal compression sampling.

**Figure 2:**
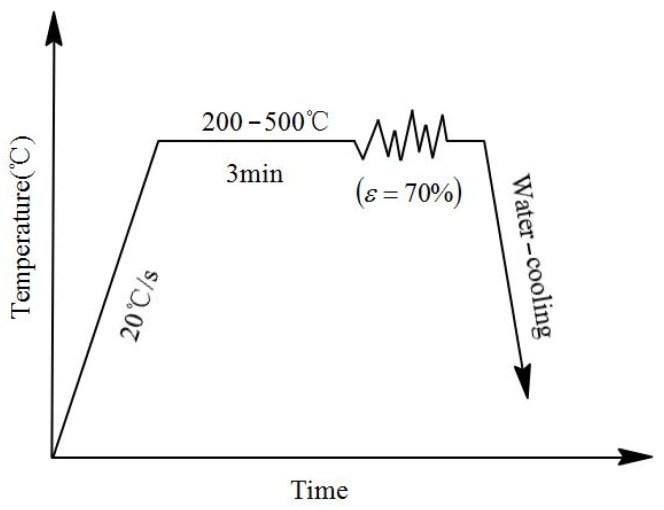
Flowchart of thermal compression of the Cu-Sn-P alloy work piece.

The experimental material was as-forged Cu-Sn-P alloy, the chemical composition of which is listed in Table 1. As shown in Figure 1, samples of 8 mm in diameter and 12 mm in height, were subjected to 70% height reduction on 3800 Gleeble at 200–500 °C and a strain rate of 0.5 s^−1^. Temperature increased at a rate of 20 °C/s and was maintained for 5 min (Figure 2). This ensured consistent temperature difference between the inside and outside of all specimens which were subsequently subjected to single-path compression deformation. After compression, the samples were quenched in water at room temperature to maintain the hot deformed microstructures.

The EBSD experiment on the compressed samples was carried out. The electrolytic polishing solution used was 70% phosphoric acid and 30% distilled water. Electropolishing was performed at an accelerating voltage from 15 to 20 kV and a scanning step of 0.06 mm. The ZEISS ULTRA 55 FE-SEM was used as the electron microscope, and the Oxford HKL EBSD Channel 5 system was used for orientation mapping analysis. X-ray diffraction (XRD) was used to perform the phase analysis. Transmission electron microscope (TEM) observation was performed using H-800 TEM. Samples were prepared as follows. Thin foils of 60 μm in thickness were tested by transmission electron microscope (TEM), and then Electro polished by the two-jet method. The operating voltage of TEM was 210 kv (JEM-2100). The solution used to polish and etch the surfaces of bar samples was 30 vol.%HCl + 20 vol.%FeCl_3_ + 60 vol.%H_2_O.

## 3. Results and Discussion

### 3.1. Strain Hardening and DRX

**Figure 3:**
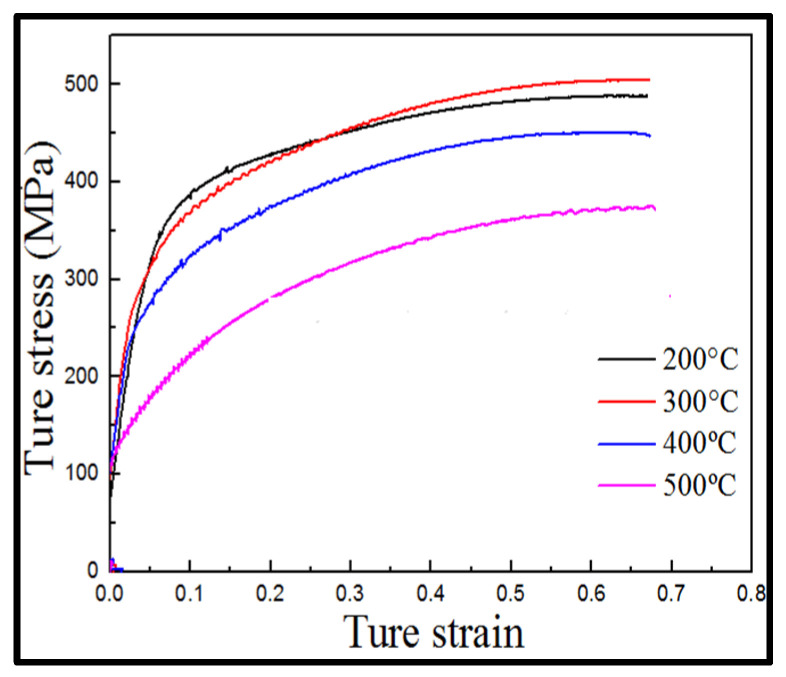
Curves showing true stress–true strain distribution for different temperatures.

In this study, a deformation strain of 70% deformation degree provided sufficient storage energy within the sample, as well as sufficient driving force for the microstructure evolution [8]. Figure 3 shows the true stress–true strain curve of single-pass compression. With increasing temperature, the flow stress decreased and the peak stress point gradually shifted to lower values. From the true stress–true strain curve, it can also be noticed that flow stress dropped quickly in the temperature range 400–500 °C, which may be due to the DRX. Since strain hardening is the dominant mechanism during hot compression, flow stress increased with strain and no steady stage appeared in the stress–strain curve. This finding was consistent with the phenomenon of “discontinuous recrystallization” in the DRX phenomenological theory that the recrystallization period is shorter than the work hardening period [8].

An uncommon observation was that when the true strain was 60%, the true stress curve of the sample deformed at 300 °C was higher than that of the sample deformed at 200 °C. According to the strain hardening theory, when strain accumulates to a certain level, the dislocation density inside the materials passes through two opposite processes, a hardening process caused by dislocation multiplication and a recovery process resulting from mutual annihilation of moving dislocations [13]. True stress–true strain curves at 200–300 °C indicated that strain hardening played a dominant role, and therefore, DRX was weak. Predictably, the 300 °C temperature could quickly promote dislocation multiplication and provide sufficient energy to drive the movement of dislocations. As the strain continued to accumulate, dislocation density increased. 

### 3.2. Microstructural Transformation Pattern of Grains

**Figure 4:**
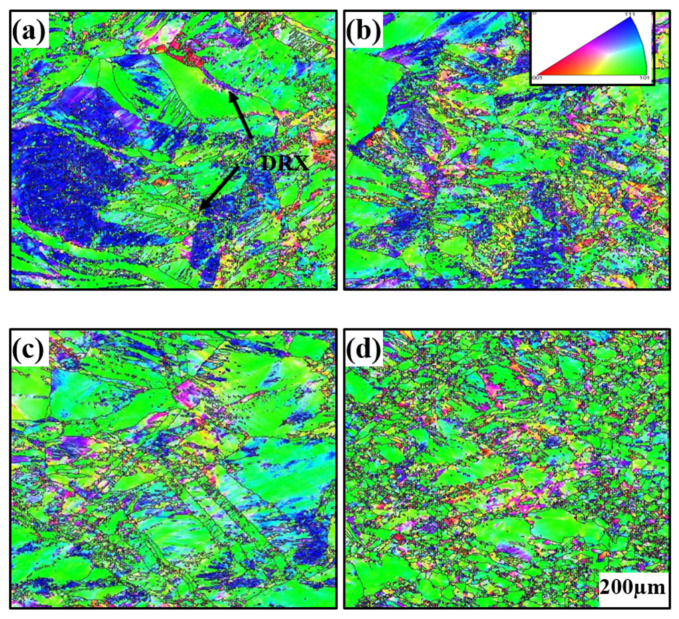
Maps showing grain orientation distribution at different temperatures: (**a**) 200 °C, (**b**) 300 °C, (**c**) 400 °C, and (**d**) 500 °C.

EBSD micrographs of the alloys after hot deformation are shown in Figure 4. Different colors represent different grain directions. Grains normal to the compression direction (CD) are elongated. The grains elongated severely with increasing deformation temperatures. Recrystallized grains were observed to be small in size, and large grains were generally deformed. For microstructural transformation of grains, incomplete recrystallization occurred inside all four compressed samples. The recrystallization grains were finer at higher compression temperatures. Another effect of increasing temperature was that the boundaries of deformed grains displayed “wavy” fluctuations because of recrystallization nucleation positions, indicating that the deformation promoted grain boundaries to attain a non-equilibrium state when grains gradually became fine, as shown in Figure 4b. When large deformed grain boundaries became flat and straight, there was evident growth of the recrystallization grains, as shown in Figure 4d.

**Figure 5:**
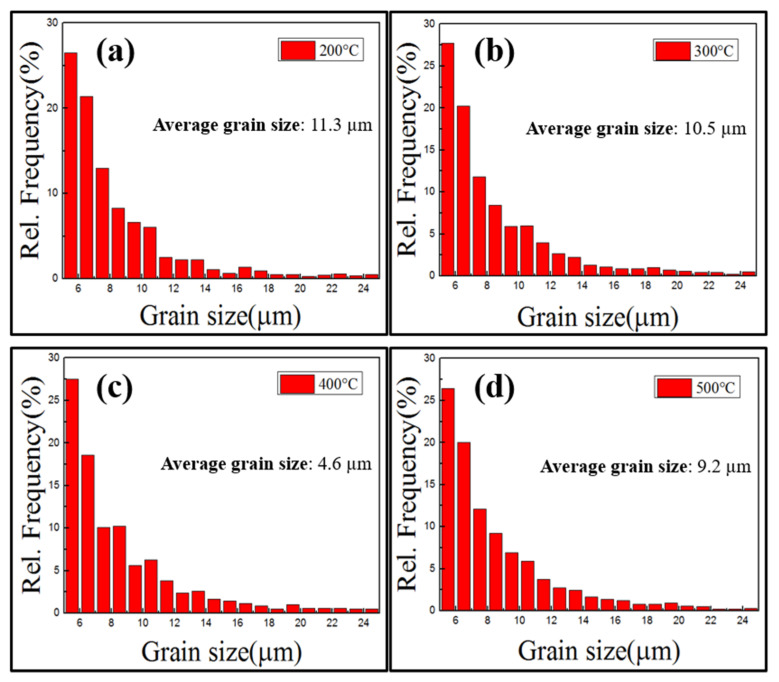
Maps showing grain size distribution at different temperatures: (**a**) 200 °C, (**b**) 300 °C, (**c**) 400 °C, and (**d**) 500 °C.

Statistics of grain size distribution are shown in Figure 5. In the 200–300 °C interval, deformation of large grains was dominant and grain size changes were miniscule. At the critical temperature of 400 °C small-sized grains gradually increased, which caused the average grain size to quickly decrease to 4.6 µm, as DRX gradually became active and the softening effect was evident. This also indicated that the recrystallization peak of the true stress–true strain curve moved leftward. For grain orientation changes, green orientations indicate the majority of the deformed grains that were not recrystallized. The {110} surfaces of such deformed grains were perpendicular to the compressive stress direction. During uniaxial compression, the orientation of the deformed grains rotated relative to the stress coordinate system. 

**Figure 6:**
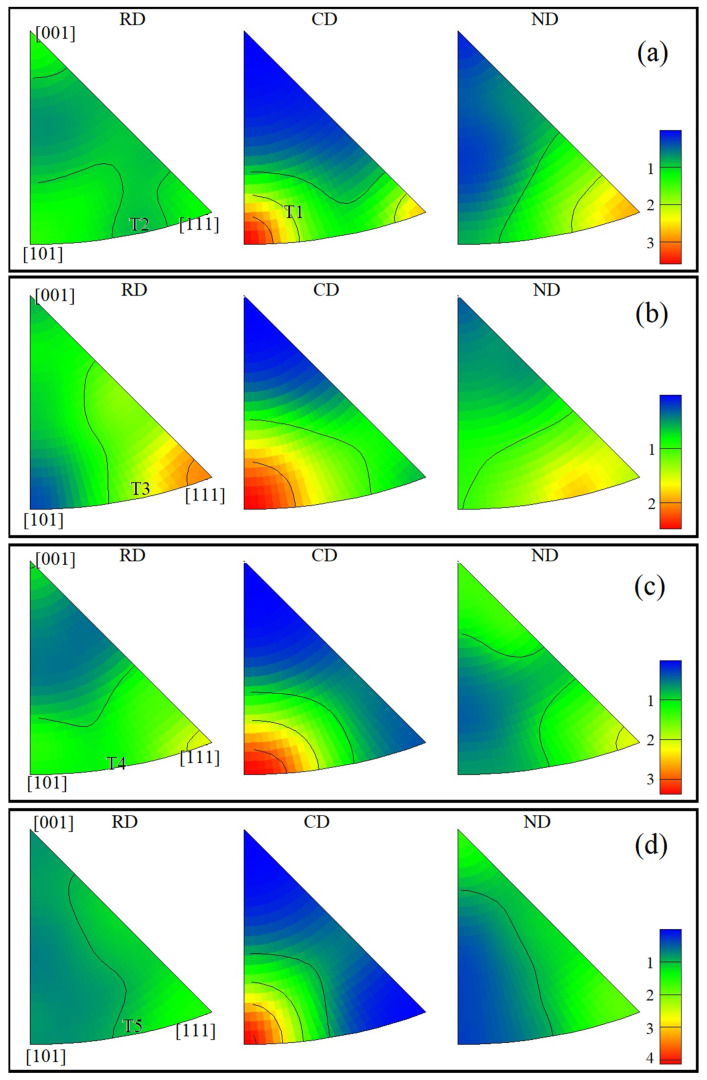
Inverse pole figures of the Cu-Sn-P alloy at different temperatures: (**a**) 200 °C, (**b**) 300 °C, (**c**) 400 °C, and (**d**) 500 °C. In the figure: radial direction (RD), compression direction (CD), and normal direction (ND).

Combined with the inverse pole figure (Figure 6), the polar density values of the textures were low at each temperature. The maximum value, 4.74, appeared at 500 °C, as shown in Figure 6d. In Figure 6a, the relatively low compression temperature and large amounts of grain deformation resembled rolling microstructures. Moreover, the recrystallization effect produced <101> sheet textures (T1) and <10> fiber textures (T2) on (111) [10$\bar{1}$] that were parallel to CD. As temperature increased, grain deformation gradually decreased and sheet textures began to disappear. At 300–500 °C, <10$\bar{1}$> (T3), <10$\bar{1}$> (T4), <101> (T5) fiber textures parallel to CD appeared on (111) [$\bar{1}$01]. In general, the typical {111} surface fiber textures of face-centered cubic (FCC) crystals were formed.

**Figure 7:**
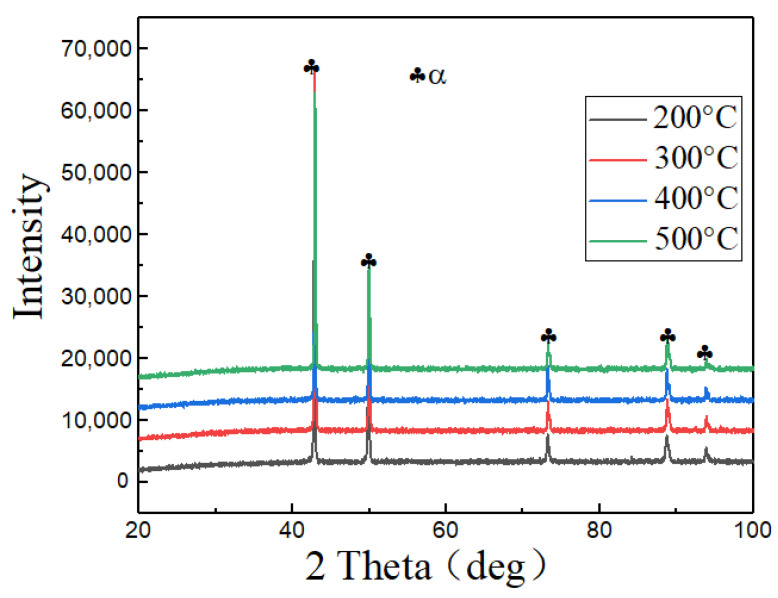
XRD patterns of the Cu-Sn-P alloy at different temperatures.

As shown in Figure 7, phase analysis of the hot compressed microstructures at different temperatures was performed using XRD. All curves had five peaks with a consistent trend of changes, and no second phase was observed. The microstructures were the single-phase Cu-based solid solution phase, which is a typical example of FCC. This also demonstrated that the hot deformation mechanism was mainly correlated with the movement of grain microstructures and dislocations and that reinforcement of the second phase was not involved.

### 3.3. Distribution Pattern of Grain Boundaries

**Figure 8:**
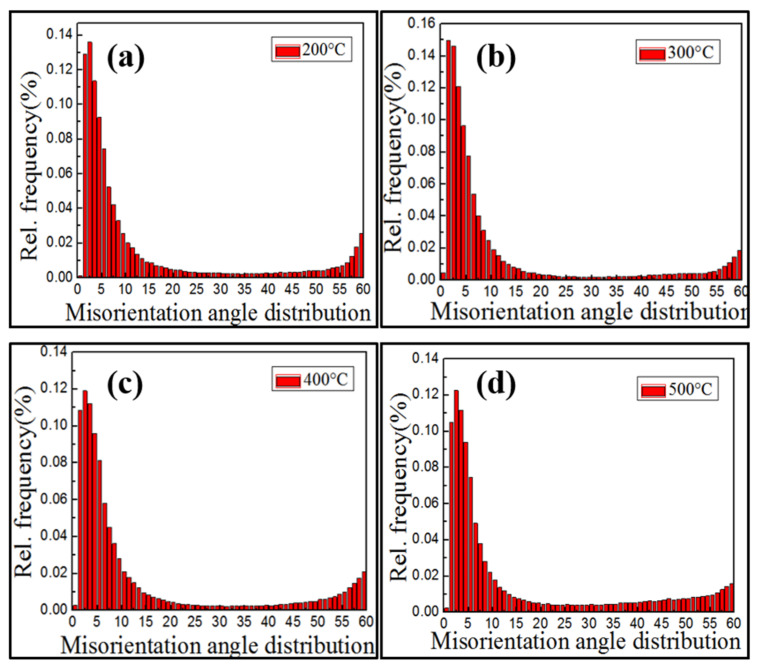
Misorientation distribution of the Cu-Sn-P alloy at different temperatures: (**a**) 200 °C, (**b**) 300 °C, (**c**) 400 °C, and (**d**) 500 °C.

Figure 8 shows the angular distribution of grain boundaries. Grain boundary orientation in hot compressed microstructures at different temperatures were mainly distributed in the ranges of 2–10° and 55–60°, exhibiting “double peaks” of the angular distribution of orientation differences. For convenience, the distribution frequency of orientation differences of each angle under different amounts of deformation in Figure 8 was measured and is presented in Table 2. Figure 8 and Table 2 indicate that deformed grains dominated HABs (≥15°) in samples at different compression temperatures. Most deformed grains were LABs (≤5°) formed by dislocation climbing and cross slip. Moreover, strain-induced LABs, formed by the excitation from bulging [8,14] on HABs of deformed grains, connected with original LABs, which further promoted the formation of sub-grains. The massive number of sub-grains led to more strain concentration inside grains. Comparison between Figure 8a,b reveals that as temperature increased, the number of low-angle sub-grain boundaries grew with more concentrated dislocations. From the perspective of grain boundary, the results indicated the true stress of the 300 °C curve surpassed the true stress of the 200 °C curve, as described in Section 3.1. As the hot compression temperature increased, HABs slowly increased. In Figure 8c,d, the number of LABs decreased significantly more than that at 200–300 °C. Major changes occurred because of the further merging and growth of HABs, or the grain boundary of the DRX grains slipping.

### 3.4. Changing Patterns of Matrix Microstructures under TEM

**Figure 9:**
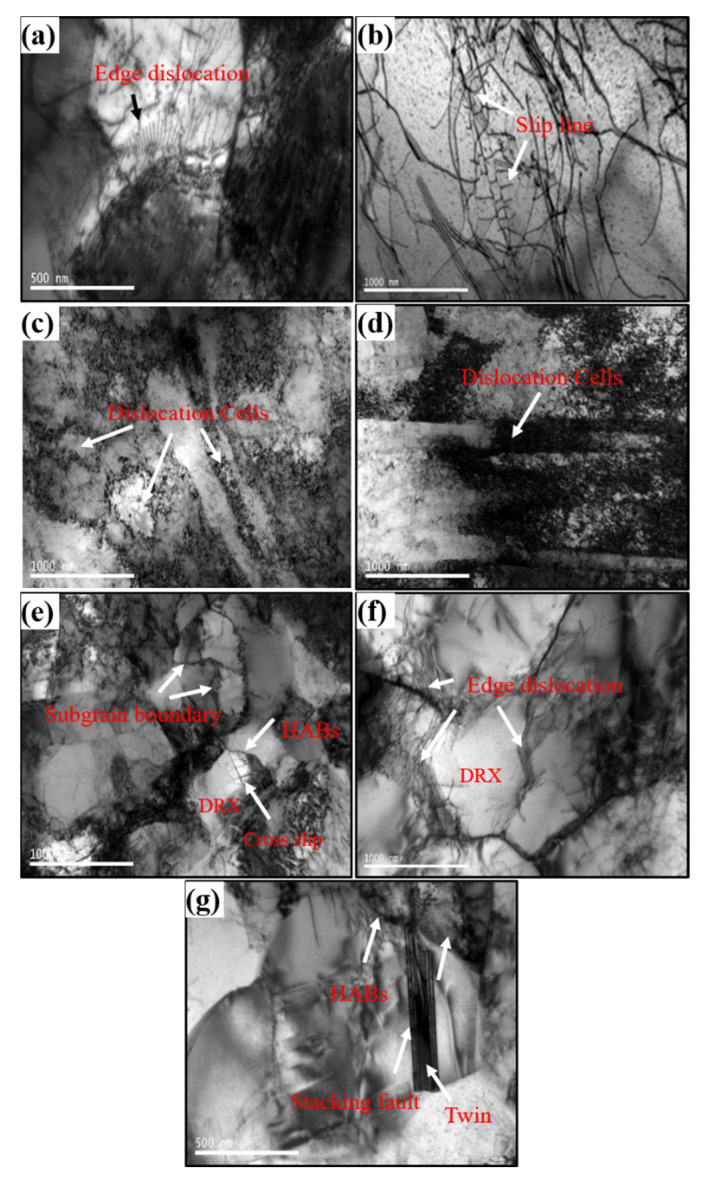
TEM pictures of the Cu-Sn-P alloy at different temperatures: (**a**,**b**) 200 °C, (**c**,**d**) 300 °C, (**e**) 400 °C, and (**f**,**g**) 500 °C.

Figure 9 shows TEM images at different temperatures. Hot deformation resulted in high density of dislocations, and the reinforcing effect of dislocations was the major resistance to plastic deformation of the material. For example, in Figure 9a, at 200 °C, the dislocation configuration was characteristic of massive tangled dislocations, when short-range interactions occur between the dislocations, and pile-up at grain boundaries strengthening the material significantly. Dislocation sources initiate dislocations at grain boundaries where dislocations pile up, which form edge dislocation arrays that slip along the grains. The dislocations were mutually parallel, and the spacing of the dislocation arrays gradually increased as they continued slipping. In the case of densely distributed dislocation arrays, it is difficult to observe the slip bands. To tackle this, this study chose to observe the inside of the deformed grains with fewer dislocations at the same temperature, such as in Figure 9b, and ended up discovering the traces of dislocation slip lines. As temperature was increased to 300 °C, the driving force for dislocations increased. Dislocation configuration transformed to dislocation bands parallel to each other near the grain boundaries, due to the grains gradually rotating in the direction perpendicular to, or forming a certain angle with, the CD at 300 °C. When this occurred, dislocation cells formed due to the entanglement of highly dense dislocations inside the grains. The dislocation bands and dislocation cells emerged because of the clustering of dislocations under interaction. Dislocation clustering significantly offsets the interaction that causes such clustering in the first place. The dislocation pile-up in Figure 9c,d was more severe, and it enhanced the hardening effect. When temperature was increased to 400 °C, as shown in Figure 9e, dislocation clustering continued to form dislocation cells. However, dislocation bands gradually vanished in dislocation slipping at high temperatures and formed dislocation cells. In this process, the high-stress fields of piled-up groups of dislocation are less stressful, as all dislocation cells slowly merge and grow as the sub-grain boundaries migrate [15,16,17]. High temperature resulted in recrystallized HABs migrating to dislocation-dense high-stress zones, as shown in Figure 9e, leaving traces of cross slipping dislocations inside recrystallized grains. As shown in Figure 9f, when temperature reached 500 °C, dislocations accumulated at grain boundaries, and there were no dislocations in DRX grains. Interestingly, step-like annealing twins were observed to be growing at 500 °C, as shown in Figure 9g. They grew in the same direction as the recrystallized HABs migrated, which is indicated by the white arrow in Figure 9g. Stacking faults existed in annealing twins that grew because of stacking fault bundles (also known as thin twins) constantly combining to form twins, rather than twinning dislocations growing under the polar axis mechanism [18,19].

## 4. Conclusions

The polar density of textures gradually increases because of the distributed fiber textures along the (111) plane as temperature increases. Sub-grains and dislocation clustering zones are mainly distributed in the hot deformed sample. At 500 °C, step-like stacking fault bundle annealing twins appear in the dynamic recrystallized grains. The main conclusions from the present study are as follows:

(1) The Cu-Sn-P alloy is strain hardened during hot deformation, accompanied with recrystallization softening alternatively taking effect. Changes in dislocation density, textures, and grain sizes play different roles in flow stress behaviors of Cu-Sn-P alloy, and the dislocation density has a more evident effect. At higher temperatures, recrystallization softening gradually becomes the dominant factor.
(2) LABs account for the majority of hot deformed microstructures. High dislocation density in this zone makes it easy to initiate the slipping systems. Deformation is realized through dislocation slips and the slip of edge dislocation pairs. Dislocation pile-up zones have high distortion energy and elements that are easy to diffuse because of which these zones are prone to recrystallization nucleation. At different temperatures, the maximum polar density of textures gradually increases, and there are preferred orientations of grains.
(3) At 500 °C, stacking faults accumulate and promote the growth of twinning. The twin growth direction is mainly determined by the migration of HABs and the clustering of high-stress zones.

## Figures and Tables

**Table 1:** Chemical composition of the Cu-Sn-P alloy (wt. %).

Sn	Al	Bi	Fe	Si	Sb	P	Cu
7.5	0.002	0.002	0.05	0.002	0.002	0.2	Bal

**Table 2:** Frequency of grains with various misorientations in the Cu-Sn-P alloy.

Misorientation	Frequency (%)	Frequency (%)	Frequency (%)	Frequency (%)
Deg	200 °C	300 °C	400 °C	500 °C
<5°	0.47	0.52	0.44	0.44
5–15°	0.30	0.29	0.32	0.27
>15°	0.23	0.19	0.24	0.29
